# P-426. Contezolid in Pediatric Hematology Patients: Efficacy and Safety in Complicated Skin and Soft Tissue Infections

**DOI:** 10.1093/ofid/ofaf695.642

**Published:** 2026-01-11

**Authors:** Yu Zhang, Xiaodong Wang, Chunjing Wang, Qian Zhang, Yue Li, Chunlan Yang, Xiaohui Zhou, Tao Chen

**Affiliations:** Shenzhen Children's Hospital, Shenzhen, Guangdong, China; Shanghai Children’s Medical Center, Shanghai, Shanghai, China; Shenzhen Children's Hospital, Shenzhen, Guangdong, China; Shenzhen Children's Hospital, Shenzhen, Guangdong, China; Shenzhen Children's Hospital, Shenzhen, Guangdong, China; Shenzhen Children's Hospital, Shenzhen, Guangdong, China; Shenzhen Children's Hospital, Shenzhen, Guangdong, China; Shanghai Institute of Infectious Disease and Biosecurity, Fudan University, Shanghai, Shanghai, China

## Abstract

**Background:**

Children undergoing chemotherapy or bone marrow transplantation face a high risk of complicated skin and soft tissue infections, predominantly caused by drug-resistant Gram-positive bacteria. While vancomycin and linezolid are effective treatments, they carry risks of renal damage and bone marrow suppression. Contezolid, a novel oxazolidinone, shows promise in adult infections but lacks pediatric data. This study evaluates the efficacy and safety of oral contezolid in pediatric hematology patients with such infections.

**Table 1.**
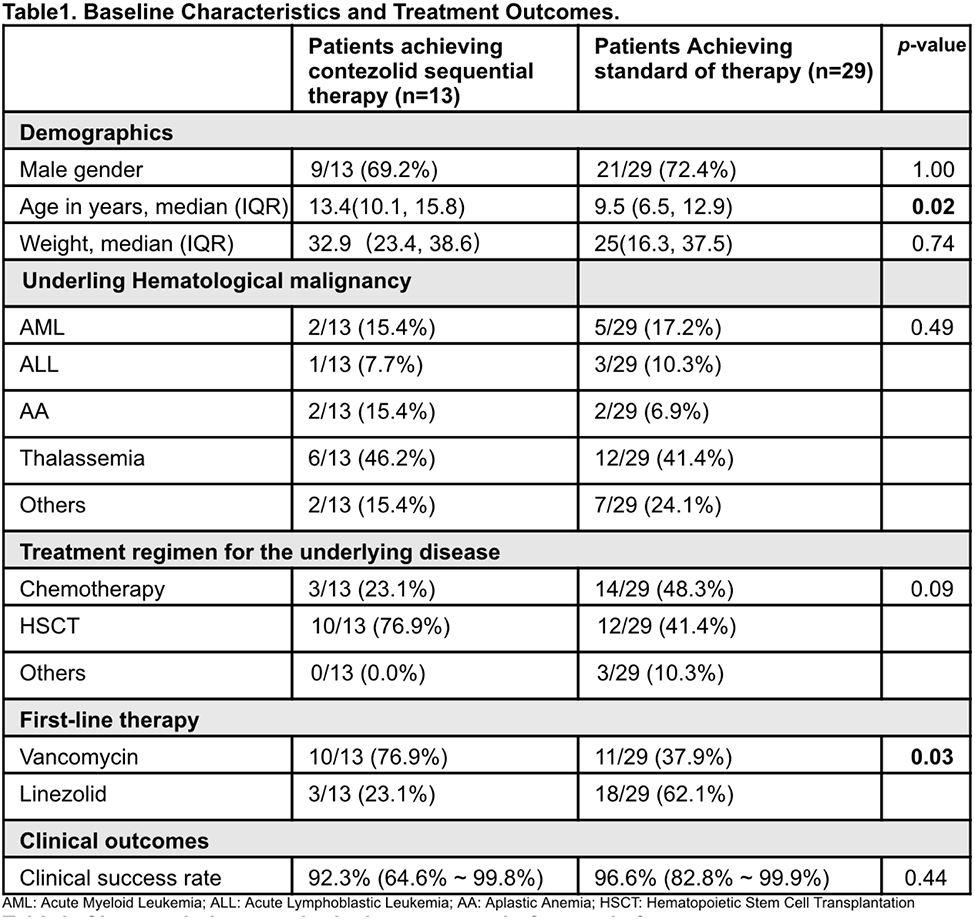
Baseline Characteristics and Treatment Outcomes.

**Table 2.**
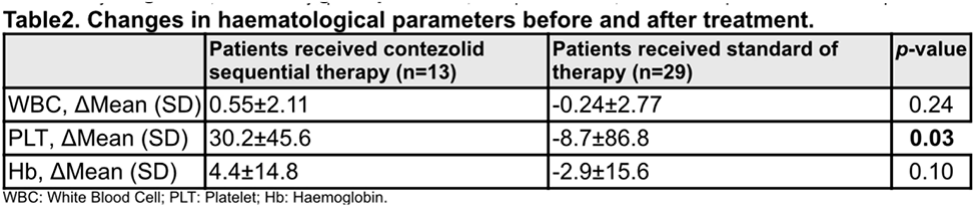
Changes in hematological parameters before and after treatment.

**Methods:**

A retrospective analysis was performed on cases from the Hematology Department at Shenzhen Children's Hospital between February 2022 and March 2024. The study enrolled patients under 18 with hematological diseases who developed post-treatment complicated skin and soft tissue infections and received either standard therapy (vancomycin or linezolid) or sequential contezolid treatment for over 3 days. Statistical analyses, including t-tests, paired t-tests, Mann-Whitney non-parametric tests, chi-square tests, and Fisher's exact probability method, were used.

**Results:**

Among 42 patients, 13 (31.0%) received contezolid, and 29 (69.0%) received standard treatment (Table 1). The contezolid group was older (median 13.4, IQR 10.1 - 15.8 vs. 9.5, IQR 6.5 - 12.9, p = 0.02), and had different first-line treatments: 76.9% vancomycin and 23.1% linezolid in the contezolid group vs. 37.9% vancomycin and 62.1% linezolid in the standard group (p = 0.03). Underlying hematological disease prevalence was similar. In the contezolid group, all patients switched to oral contezolid (200 - 800mg bid) after stabilization. Clinical cure rates were 92.3% (64.6% - 99.8%) and 96.6% (82.8% - 99.9%) for contezolid and standard groups, respectively (p = 0.44). No significant treatment-related adverse events occurred. Hematological analysis showed contrasting trends between groups, with a significant difference in platelet count change (30.2 ± 45.6 vs. -8.7 ± 86.8, p = 0.03) (Table2).

**Conclusion:**

Oral contezolid exhibits good efficacy and tolerability in pediatric hematology patients, potentially offering hematological safety advantages. Further research is needed to validate its clinical benefits in childre

**Disclosures:**

Xiaodong Wang, Doctor of Pediatrics, MicuRx: Grant/Research Support Tao Chen, Master of pediatrics, MicuRx: I am an employee of MicuRx

